# Modeling and experimental analysis of CO_2_ methanation reaction using Ni/CeO_2_ monolithic catalyst

**DOI:** 10.1007/s11356-024-33327-5

**Published:** 2024-04-25

**Authors:** Adriana Parra-Marfil, Raúl Ocampo-Pérez, Carlos Gilberto Aguilar-Madera, Francisco Carrasco-Marín, Agustín Francisco Pérez-Cadenas, Agustín Bueno-López, Esther Bailón-García

**Affiliations:** 1 Materiales Polifuncionales Basados en Carbono (UGR-Carbon), Dpto. Química Inorgánica - Unidad de Excelencia Química Aplicada a Biomedicina y Medioambiente, Universidad de Granada (UEQ-UGR), 18071 Granada, ES Spain; 2 Centro de Investigación y Estudios de Posgrado (CIEP), Facultad de Ciencias Químicas, Universidad Autónoma de San Luis Potosí (FCQ-UASLP), 78260 San Luis Potosí, MX Mexico; 3 Facultad de Ciencias de La Tierra, Universidad Autónoma de Nuevo León (UANL), Carretera a Cerro Prieto Km. 8 Ex Hacienda de Guadalupe, 67700 Linares, MX Mexico; 4 Dpto. de Química Inorgánica, Universidad de Alicante (UA), 03080 Alicante, ES Spain

**Keywords:** Sabatier reaction, CO_2_ valorization, Fluid dynamics, Mathematical modeling

## Abstract

In this study, the effect of the cell density of monolithic catalysts was investigated and further mathematically modeled on cordierite supports used in CO_2_ methanation. Commercial cordierite monoliths with 200, 400, and 500 cpsi cell densities were coated by immersion into an ethanolic suspension of Ni/CeO_2_ active phase. SEM–EDS analysis confirmed that, owing to the low porosity of cordierite (surface area < 1 m^2^ g^−1^), the Ni/CeO_2_ diffusion into the walls was limited, especially in the case of low and intermediate cell density monoliths; thus, active phase was predominantly loaded onto the channels’ external surface. Nevertheless, despite the larger exposed surface area in the monolith with high cell density, which would allow for better distribution and accessibility of Ni/CeO_2_, its higher macro-pore volume resulted in some introduction of the active phase into the walls. As a result, the catalytic evaluation showed that it was more influenced by increments in volumetric flow rates. The low cell density monolith displayed diffusional control at flow rates below 500 mL min^−1^. In contrast, intermediate and high cell density monoliths presented this behavior up to 300 mL min^−1^. These findings suggest that the interaction reactants-catalyst is considerably more affected by a forced non-uniform flow when increasing the injection rate. This condition reduced the transport of reactants and products within the catalyst channels and, in turn, increased the minimum temperature required for the reaction. Moreover, a slight diminution of selectivity to CH_4_ was observed and ascribed to the possible formation of hot spots that activate the reverse water–gas shift reaction. Finally, a mathematical model based on fundamental momentum and mass transfer equations coupled with the kinetics of CO_2_ methanation was successfully derived and solved to analyze the fluid dynamics of the monolithic support. The results showed a radial profile with maximum fluid velocity located at the center of the channel. A reactive zone close to the inlet was obtained, and maximum methane production (4.5 mol m^−3^) throughout the monolith was attained at 350 °C. Then, linear streamlines of the chemical species were developed along the channel.

**Supplementary Information:**

The online version contains supplementary material available at 10.1007/s11356-024-33327-5.

## Introduction

The carbon dioxide conversion to value-added products appears as a promising alternative to reduce the environmental problems caused by CO_2_-dominated greenhouse gases. Several techniques have been employed for CO_2_ reduction, including thermochemical (Shuai et al. 2021; Amica and Gennari 2022; González-Castaño et al. 2023), biochemical (Nisar et al. 2021; Sharma et al. 2022), electrochemical (Albo et al. 2019; Oßkopp et al. 2022; Wang et al. 2022; Song et al. 2022), and photochemical (Kuramochi et al. 2018; Yaashikaa et al. 2019; Pashchenko 2022). Among the technologies mentioned above for CO_2_ valorization, the thermochemical transformation through catalytic hydrogenation to methane has gained attention since it is a strategy that can treat two environmental issues at a time: the accumulation of greenhouse gases in the atmosphere and the energy demand (Chen et al. 2021a; Fan and Tahir 2021; Shewchuk et al. 2021; Cordero-Lanzac et al. 2023).

The catalytic CO_2_ hydrogenation to methane, also known as CO_2_ methanation or Sabatier reaction (Eq. 1), is a high exothermic equilibrium reaction (Navarro et al. 2018; Renda et al. 2021).

1 $${{\mathrm{CO}}}_{2}+4{{\mathrm{H}}}_{2}\leftrightarrow {{\mathrm{CH}}}_{4}+2{{\mathrm{H}}}_{2}{\mathrm{O}},\Delta {{\mathrm{H}}}_{298{\mathrm{K}}}=-164.9{{\mathrm{kJmol}}}^{-1}$$

This process has several environmental implications such as the following: (i) carbon sequestration since CO_2_ methanation offers a potential pathway for carbon capture and storage (CCS) by converting CO_2_ into methane helping to mitigate climate change; (ii) utilization of CO_2_: methanation provides a means of utilizing CO_2_ as a feedstock for the production of methane, an useful fuel or chemical feedstock, rather than allowing them to be released directly into the atmosphere; and (iii) renewable energy storage: methane produced through CO_2_ methanation can be used as a renewable energy storage medium. It can be stored and transported through existing natural gas infrastructure (Chen et al. 2021b) providing a means of storing excess renewable energy generated from sources such as wind and solar power for use during periods of low generation. Overall, while CO_2_ methanation offers potential environmental benefits such as carbon sequestration and renewable energy storage, its environmental implications depend on factors such as the source of hydrogen, energy efficiency of the process, methane leakage, and land use impacts. Therefore, careful assessment and management of these factors are necessary to maximize the environmental benefits of CO_2_ methanation while minimizing its potential negative impacts.

The Sabatier reaction is thermodynamically favored at low temperatures; however, it is kinetically limited due to the high carbon dioxide stability and hydrogen activation (Chen et al. 2021b). Therefore, stable and efficient catalysts are needed to improve the reaction conditions and increase the reaction rate of CO_2_ methanation. In this sense, catalysts based on Ni species supported on different oxides (namely CeO_2_, Al_2_O_3_, TiO_2_, SiO_2_, ZrO_2,_ and so on) are the most commonly used in industrial scale methanation processes (Frontera et al. 2017; Chen et al. 2021a; Gómez et al. 2022). Regarding other metals, metallic nickel results are attractive because of its high catalytic activity, high methane selectivity, and relatively low cost (De Piano et al. 2022; Gómez et al. 2022). On the other hand, among the previously mentioned supports, CeO_2_ is recognized to promote CO_2_ methanation, enhancing CH_4_ formation. Also, CeO_2_ improves the metal-support interaction resulting in an excellent low-temperature catalytic performance (Laosiripojana and Assabumrungrat 2006; Prakash et al. 2007). Nevertheless, during exothermic CO_2_ methanation, the temperature increases, forming hot spots and leading to catalyst deactivation by Ni particles agglomeration/sintering and carbon deposition (Zhang et al. 2023). Hence, the catalyst structure has an essential role in enabling good heat and mass transfer inside the reactor to get high stability and activity.

Compared to packed-bed systems, structured systems using monolithic catalysts present several advantages, such as better heat exchange and fluid processing and a significant reduction in pressure drop, allowing to treat high feed flows. Moreover, using monolithic catalysts also results in easier manipulation, separation, and cleaning processes at an industrial scale. Typically, monolithic structures used for supporting catalytic active phases are either ceramic or metallic (Forzatti et al. 1998; Colman-lerner et al. 2016; Kucharczyk et al. 2017; Quan et al. 2018). In that regard, few studies have been developed employing monolithic structures supporting catalytic active phases for CO_2_ methanation. Fukuhara et al. (2017) prepared a CO_2_ methanation catalyst by washcoating an aluminum honeycomb monolith with a Ni/CeO_2_-propanol suspension. The catalyst produced showed high activity and selectivity owing to better mass and heat transfer properties. Danaci et al. (2016) investigated the performance of a stainless-steel monolithic structure coated with Ni/Al_2_O_3_ for CO_2_ methanation. The results showed improvement using the metallic support compared to the powder catalyst (an increase of CO_2_ conversion near to 90%, and stable methane selectivity at around 95–99%). Nonetheless, they have noticed the importance of the slurry properties in coating adhesion on metallic supports, for which the addition of acid to avoid agglomeration of particles and binder to provide suitable viscosity and suspension stability was needed. Hence, the requirement and influence of surface pretreatments and/or coating additives on the catalytic properties are a disadvantage of this type of material.

Ceramic materials, among which cordierite is the most widely used, offer high thermal and chemical stability and better active phase adherence in comparison to metallic ones (Al-Harbi et al. 2015; Pratap et al. 2020; Chaparro-Garnica et al. 2022). In this context, NiFe catalyst was prepared on alumina-washcoated cordierite monolith by Huynh et al. (2020), which allowed them to treat high flow rate (500 mL min^−1^), achieving great methane yield from CO_2_ methanation. Besides, Vita et al. (2020) studied the behavior of a cordierite monolith loaded with 25 wt% Ni/GDC (gadolinium-doped-ceria) towards the conversion of CO_2_ to CH_4_. The catalyst facilitated the temperature distribution, thus avoiding coking and sintering, along with enabling high space velocity operation with great stability and CO_2_ conversion (~ 70%). Furthermore, other configuration parameters, such as hydrodynamic design, are also determining for its usefulness in selective oxidation processes (Vega et al. 2021; García-Moncada et al. 2022). With an increase in cell density, a larger surface to volume ratio is gained, leading to a greater amount of deposited and accessible active phase, and, therefore, a higher CO-PROX activity is expected (Barbato et al. 2015). However, some authors have observed that dynamic performance is deteriorated due to a mass transfer inhibition by molecular diffusion in VOC abatement or adsorption kinetics (Santos et al. 2020; Lawson et al. 2020). However, a lack of information exists about the behavior of cordierite monoliths and the effect of cell density, flow rates, and the active phase distribution along the monolith porosity on the catalytic performance for the CO_2_ methanation.

Considering this, the present work aims to evaluate the performance of Ni/CeO_2_-coated cordierite monoliths in the Sabatier reaction as a way for improving the catalytic activity in the treatment of high CO_2_ flow rates. Moreover, different cell densities (200, 400, and 500 cpsi) were employed to study the influence of monolith configuration on CO_2_ conversion and selectivity to CH_4_. Additionally, the current study establishes an appropriate mathematical model to analyze the fluid dynamics of the materials, as well as to predict the velocity profiles inside the channels of the catalytic structure used. Therefore, the understanding achieved in this work is very promising for the development and improvement of monolithic catalysts for CO_2_ methanation and works towards achieving the sustainability and environmental protection goals.

## Experimental

### Catalyst preparation

#### Active phase synthesis

Firstly, the CeO_2_ was obtained by thermal decomposition of cerium nitrate hexahydrate (Ce(NO_3_)_3_·6H_2_O, ≥ 99.5% from Alfa Aesar) by calcination in a muffle at 500 °C for 4 h. Then, the Ni/CeO_2_ active phase was prepared by incipient wetness impregnation of the CeO_2_ with an ethanolic solution of nickel (II) nitrate hexahydrate (Ni(NO_3_)_2_·6H_2_O, ≥ 97% Sigma-Aldrich) to attain 10 wt% of Ni. Subsequently, the impregnated CeO_2_ was calcined at 600 °C for 5 h to decompose the salt and obtain the final Ni/CeO_2_ active phase.

#### Active phase loading

**Fig. 1:**
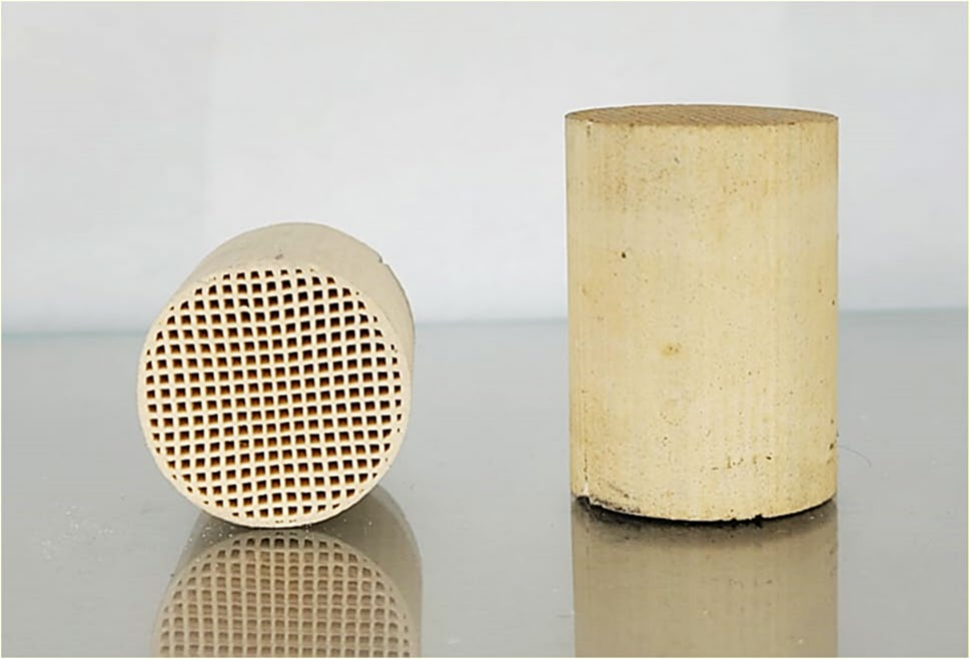
Commercial honeycomb cordierite monoliths used for evaluation of CO_2_ methanation

The previously obtained Ni/CeO_2_ was deposited onto commercial cordierite monoliths with three different cell densities (200, 400, and 500 cpsi, named CoL, CoI, and CoH, correspondingly) using the dip-coating method. These cylindrical honeycomb cordierite monoliths, of approximately 2.3 cm in diameter and 3.5 cm in length, are composed by straight channels with a square cross-section (Fig. 1) and were supplied by CTI (Céramiques Techniques et Industrielles; France). The monoliths were immersed into a Ni/CeO_2_ ethanolic suspension (10 wt%). Then, they were dried for 12 h at room temperature while rotating horizontally to obtain homogeneous distribution of the Ni/CeO_2_, and later at 80 °C overnight. The exceeding active phase not anchored to the monoliths that can block the channels was dropped using compressed air. The amount of Ni/CeO_2_ loaded was corroborated by the weight difference of the dried monoliths after each impregnation, and the process was repeated until achieving 800 ± 5 mg of Ni/CeO_2_ per monolith.

### Catalyst characterization

To get information about the surface area of the Ni/CeO_2_ powder catalyst, adsorption isotherms (N_2_ at − 196 °C) were obtained employing a Quantachrome Autosorb-6B equipment. The samples were degassed at 110 °C for 8 h before the gas adsorption measurements. The surface chemistry of the active phase Ni/CeO_2_ and the CeO_2_ support was analyzed using X-ray photoelectron spectroscopy (XPS) with a K-ALPHA Thermo Scientific device. The X-ray source utilized was Al-Kα radiation at 1486.6 eV. Besides, its crystalline structure was studied by X-ray diffraction (XRD) and Raman spectroscopy. Raman analysis was done by a JASCO NRS-5100 dispersive micro-Raman microscope with a Green Diode 532 nm, 30 mW (Elforlight G4-30; Nd:YAG). The XRD patterns of the catalyst were measured using an X-Ray Diffractometer Bruker D8 DISCOVER with CuKα-ray radiation (*λ* = 1.54178 Å) in the 2*θ* range of 10–90° at a step size of 1° min^−1^. The average crystal size (*d*) was determined using the Scherrer Eq. (2) (Scherrer 1912; Burton et al. 2009).

2 $$d=\frac{K\cdot \lambda }{B\cdot {\mathrm{cos}}\theta }$$

where *d* is the crystallite size, *B* is the full width at half maximum in radians, *K* is the shape factor (0.94), $\lambda$ is the X-ray wavelength (0.15 nm), and $\theta$ is the diffraction angle in radians.

The porosity of the ceramic monoliths was analyzed by Hg-porosimetry using a Micromeritics AutoPore IV 9500 equipment. The analysis was conducted from an initial pressure of 5 psi up to 30, 500 psi, with 10 s of equilibration time between points. The contact angle and surface tension used for pore size calculations were *θ* = 130° and *γ* = 485 dyne/cm, respectively. The active phase distribution on the cordierite monoliths was analyzed by scanning electron microscopy (SEM) using a SUPRA40VP Variable Pressure Scanning Electron Microscope (VP-FESEM) from Zeiss equipped with an EDX detector X-max 50 for microanalysis and chemical mapping.

Moreover, compression tests were realized to analyze the mechanical properties of ceramic monoliths. Firstly, the monolith samples were cut into cylinders 1.5 cm high, and the bases were sanded to obtain smooth and even surfaces. The tests were carried out with a constant approach speed of 1 mm-min^−1^ utilizing a Shimadzu AGS-J 10 kN instrument, and the compression test direction was top to bottom. The corresponding compressive stress–strain curves were analyzed to obtain the Young’s modulus (*E*) and the maximum strength (*σ*) were determined from stress–strain curve analysis.

### Catalytic tests

The monolithic catalyst performance and the effect of cell density were analyzed in the methanation of carbon dioxide. A monolith was fixed in a quartz cylindrical reactor and placed in a furnace with temperature control. A cold trap was settled at the outlet of the reactor to retain the water produced, and the outflow gases CO_2_ and CH_4_ were analyzed by infrared gas sensors (Edinburgh Instruments LTD 034/00700). Prior to each experiment, the monolith to be studied was treated in a reducing atmosphere at 500 °C for 1.5 h. Afterwards, the system was let to cool down to 150 °C, so the inlet flow was changed to the reaction gas mixture (10% CO_2_, and 40% H_2_ balanced in Ar) with total flows ranging from 100 to 500 mL min^−1^. The temperature was increased up to 450 °C in steps of 25 °C, holding each temperature for 20 min. The CO_2_ conversion ($\%{\mathrm{C}}_{{\mathrm{CO}}_{2}})$ and selectivity to CH_4_ (${\mathrm{\%}{\mathrm{S}}}_{{\mathrm{CH}}_{4}})$ were calculated to evaluate the catalytic performance according to the following equations:

3 $${\mathrm{\%}\mathrm{C}}_{{\mathrm{CO}}_2}\mathrm{(\%)=}\frac{{\mathrm{F}{\mathrm{CO}}_2}_\mathrm{in}-{\,\mathrm{F}{\mathrm{CO}}_2}_\mathrm{out}}{{\mathrm{F}{\mathrm{CO}}_2}_\mathrm{in}}\mathrm{x100\%}$$

4 $$\%{\mathrm{S}}_{{\mathrm{CH}}_4}\left(\%\right)\mathrm{=}\frac{{\mathrm{FCH}}_4}{{{\mathrm{FCO}}_2}_\mathrm{in}\mathrm{-}{\mathrm{F}{\mathrm{CO}}_2}_\mathrm{out}}\mathrm{x100\%}$$

where FCO_2 in_, FCO_2 out_, and FCH_4_ refer to the volumetric flow of CO_2_ in the inlet and outlet of the reactor, and the volumetric flow of CH_4_ obtained, respectively.

### Mathematical model

The experimental data were interpreted with a mathematical model involving the mass and momentum balances for the reaction gas mixture. The mass transfer was analyzed considering the mass transport of CO_2_ and H_2_ from the bulk to the external surface of the reactive monolith walls. It is assumed that the monolith has low porosity; thus, the solute diffusion is negligible. According to the chemical reaction (1), the mathematical model includes nine partial differential equations. Three equations correspond to the momentum balance, one equation states the gas-phase mass balance, and additionally, there is one mass balance for each chemical species including argon, since, although it is a nonreactive compound, the species molar fractions depend on all the gases, and they must sum one in total. For momentum balance, we used the $\kappa -\epsilon$ mathematical model, considering the possibility of the flow developing turbulence inside the monolith for all the experimental cases.

The momentum balance is given by:

5 $$\rho \frac{\partial {\varvec{u}}}{\partial t}+ \rho \left({\varvec{u}}\bullet \nabla \right){\varvec{u}}=-\nabla p+\nabla \bullet \mathbf{K}$$

With,

6 $$\mathbf{K}=\left(\upmu +{\upmu }_{T}\right)\left[\nabla {\varvec{u}}+{\left(\nabla {\varvec{u}}\right)}^{T}\right]$$

Here, ${\upmu }_{T}$ is the turbulent viscosity computed as follows:

7 $${\upmu }_{T}=\rho {C}_{\mu }\frac{{\kappa }^{2}}{\epsilon }$$

The turbulent kinetic energy $\kappa$ obeys the following partial differential equation:

8 $$\rho \frac{\partial \kappa }{\partial t}+ \rho \left({\varvec{u}}\bullet \nabla \right)\kappa =\nabla \bullet \left[\left(\upmu +{\upmu }_{T}\frac{{\upmu }_{T}}{{\sigma }_{\kappa }}\right)\nabla \kappa \right]+{P}_{\kappa }-\rho \epsilon$$

While the rate of dissipation of turbulent kinetic energy is given by:

9 $$\rho \frac{\partial \epsilon }{\partial t}+ \rho \left({\varvec{u}}\bullet \nabla \right)\epsilon =\nabla \bullet \left[\left(\upmu +{\upmu }_{T}\frac{{\upmu }_{T}}{{\sigma }_{\epsilon }}\right)\nabla \epsilon \right]+{C}_{\epsilon 1}\frac{\epsilon }{\kappa }{P}_{\kappa }-{C}_{\epsilon 2}\rho \frac{{\epsilon }^{2}}{\kappa }$$

The term associated to the rate of deformation is defined as:

10 $${P}_{\kappa }={\upmu }_{T}\left\{\nabla {\varvec{u}}:\left[\nabla {\varvec{u}}+{\left(\nabla {\varvec{u}}\right)}^{T}\right]\right\}$$

In addition, **ρ** represents the fluid mass density, ${\varvec{u}}$ is the vector of velocity, $t$ is the time, $p$ is the pressure, and **μ** is the fluid viscosity. The gas-phase mass balance is given by the continuity equation written as:

11 $$\frac{\partial \rho }{\partial t}+ \nabla \bullet \left(\rho {\varvec{u}}\right)=0$$

The mass balance for each chemical species in terms of molar concentration, $C$, is shown in Eqs. (12–16), for which *D*_*i*_ refers to the molecular diffusivity of the *i-*compound in Ar.

12 $$For C{O}_{2}\frac{\partial {C}_{{CO}_{2}}}{\partial t}+ \nabla \bullet \left({\varvec{u}}{C}_{{CO}_{2}}\right)=\nabla \bullet ({D}_{{CO}_{2}}\nabla {C}_{{CO}_{2}})$$

13 $$For {H}_{2}\frac{\partial {C}_{{H}_{2}}}{\partial t}+ \nabla \bullet \left({\varvec{u}}{C}_{{H}_{2}}\right)=\nabla \bullet ({D}_{{H}_{2}}\nabla {C}_{{H}_{2}})$$

14 $$For C{H}_{4}\frac{\partial {C}_{{CH}_{4}}}{\partial t}+ \nabla \bullet \left({\varvec{u}}{C}_{{CH}_{4}}\right)=\nabla \bullet ({D}_{{CH}_{4}}\nabla {C}_{{CH}_{4}})$$

15 $$For {H}_{2}O \frac{\partial {C}_{{H}_{2}O}}{\partial t}+ \nabla \bullet \left({\varvec{u}}{C}_{{H}_{2}O}\right)=\nabla \bullet ({D}_{{H}_{2}O}\nabla {C}_{{H}_{2}O})$$

16 $$For Ar \frac{\partial {C}_{Ar}}{\partial t}+ \nabla \bullet \left({\varvec{u}}{C}_{Ar}\right)=\nabla \bullet ({D}_{Ar}\nabla {C}_{Ar})$$

Initial values of primary variables (molar concentrations, pressure, and velocity) used to solve numerically the model are considered as:

17 $$At t=0 {\mathrm{min}} { C}_{{CO}_{2}}={C}_{{CO}_{2}}^{0}, {C}_{{H}_{2}}={C}_{{H}_{2}}^{0}, {C}_{{CH}_{4}}={C}_{{CH}_{4}}^{0}, {C}_{{H}_{2}O}={C}_{{H}_{2}O}^{0}, {C}_{Ar}={C}_{Ar}^{0}, {\varvec{u}}=0, p=1 {\mathrm{atm}}$$

Further, boundary conditions were stated at the inlet and outlet of the monolith. For the inlet boundary, the velocity and molar concentrations are determined as:

18 $$\begin{array}{c}{C}_{{CO}_{2}}={C}_{{CO}_{2}}^{in}, { C}_{{H}_{2}}={C}_{{H}_{2}}^{in}, {C}_{{CH}_{4}}={C}_{{CH}_{4}}^{in}, {C}_{{H}_{2}O}={C}_{{H}_{2}O}^{in}, { C}_{Ar}={C}_{Ar}^{in}\\ -\boldsymbol{ }{\varvec{n}}\bullet {\varvec{u}}={u}^{in}\end{array}$$

where ***n*** is the unit normal vector pointing outward the inlet boundary. The inlet velocity *u*^*in*^ is computed through the flow area and volumetric flow rate as follows:

19 $${u}^{in}={~}^{Q}\!\left/ \!{~}_{A}\right.$$

whereas, at the outlet boundary the pressure is fixed, and the species flow is only due to advection:

20 $$\begin{array}{c}{{\varvec{n}}\bullet (D}_{{CO}_{2}}\nabla {C}_{{CO}_{2}})=0, {{\varvec{n}}\bullet (D}_{{H}_{2}}\nabla {C}_{{H}_{2}})=0, {{\varvec{n}}\bullet (D}_{{CH}_{4}}\nabla {C}_{{CH}_{4}})=0,\\ {{\varvec{n}}\bullet (D}_{{H}_{2}O}\nabla {C}_{{H}_{2}O})=0, {{\varvec{n}}\bullet (D}_{Ar}\nabla {C}_{Ar})=0, p =1\mathrm{ atm}\end{array}$$

The nonslip and impenetrability conditions are enforced for the velocity vector at the monolith surface, ${\varvec{u}}=0$. Meanwhile, at the reactive surface of the monolith, the normal fluxes of chemical species are stated as:

21 $$\begin{array}{c}{{\varvec{n}}\bullet (D}_{{CO}_{2}}\nabla {C}_{{CO}_{2}})=-{r}_{m}, {{\varvec{n}}\bullet (D}_{{H}_{2}}\nabla {C}_{{H}_{2}})=-4{r}_{m}, {{\varvec{n}}\bullet (D}_{{CH}_{4}}\nabla {C}_{{CH}_{4}})={r}_{m},\\ {{\varvec{n}}\bullet (D}_{{H}_{2}O}\nabla {C}_{{H}_{2}O})=2{r}_{m}, {{\varvec{n}}\bullet (D}_{Ar}\nabla {C}_{Ar})=0\end{array}$$

The auxiliary relations of the total molar concentration, molar fraction of species *i*, partial pressure of species *i*, and the mass density of the gaseous mixture obtained from the average molar mass $M$ are given by the following equations:

22 $${\mathrm{C = C}}_{{\mathrm{CO}}_{2}}+{\mathrm{ C}}_{{\mathrm{H}}_{2}}+{\mathrm{ C}}_{{\mathrm{CH}}_{4}}+{\mathrm{ C}}_{{\mathrm{H}}_{2}O}+{\mathrm{ C}}_{\mathrm{Ar}}$$

23 $${y}_{i}=\frac{{C}_{i}}{C}$$

24 $${p}_{i}={y}_{i}p$$

25 $$M={\sum }_{i}{y}_{i}{M}_{i}; \rho =MC$$

The molecular diffusivity coefficients *D*_*i*_ as function of the temperature (*T*) were estimated with the following correlations (temperature in K and diffusivity in cm^2^ s^−1^):

26 $${D}_{{CO}_{2}-Ar}=\frac{0.00143{T}^{1.75}}{(1){(41.8807)}^{0.5}{\left[{26.9}^{1/3}+{16.2}^{1/3}\right]}^{2}}=7.2346 x {10}^{-6}{T}^{1.75}$$

27 $${D}_{{H}_{2}-Ar}=\frac{0.00143{T}^{1.75}}{(1){(3.8383)}^{0.5}{\left[{6.12}^{1/3}+{16.2}^{1/3}\right]}^{2}}=3.8406 x {10}^{-5}{T}^{1.75}$$

28 $${D}_{C{H}_{4}-Ar}=\frac{0.00143{T}^{1.75}}{(1){(22.8894)}^{0.5}{\left[{25.14}^{1/3}+{16.2}^{1/3}\right]}^{2}}=1.0027 x {10}^{-5}{T}^{1.75}$$

29 $${D}_{{H}_{2}O-Ar}=\frac{0.00143{T}^{1.75}}{(1){(24.8318)}^{0.5}{\left[{13.1}^{1/3}+{16.2}^{1/3}\right]}^{2}}=1.2012 x {10}^{-5}{T}^{1.75}$$

The chemical reaction rate of CO_2_ methanation is described by a Hougen-Watson type expression (Chein et al. 2016):

30 $${r}_{m}={\rho }_{cat}\frac{k{K}_{{CO}_{2}}{K}_{{H}_{2}}^{4}{p}_{{CO}_{2}}{p}_{{H}_{2}}^{4}}{{\left(1+{K}_{{CO}_{2}}{p}_{{CO}_{2}}+{K}_{{H}_{2}}{p}_{{H}_{2}}\right)}^{5}}\left(1-\beta \right)$$

where ${r}_{m}$ (mol m^−3^ s^−1^) is the CO_2_ methanation reaction rate, ${\rho }_{cat}$ (kg m^−3^) is the catalyst density, and $\beta$ is the approach-to-equilibrium coefficient defined as:

31 $$\beta =\frac{{p}_{{CH}_{4}}{p}_{{H}_{2}O}^{2}}{{0.009p}_{{CO}_{2}}{p}_{{H}_{2}}^{4}{K}_{eq}}$$

Here, $k$ is the rate constant of CO_2_ methanation reaction (mol kg_cat_^−1^ s^−1^), ${p}_{{\mathrm{i}}}$ (*i* = CO_2_, H_2_, CH_4_, and H_2_O) is the partial pressure (atm) of species *i*, ${K}_{i}$ is the surface adsorption equilibrium constant for species *i* (atm^−1^), and ${K}_{eq}$ is the overall Sabatier reaction equilibrium constant (atm^−2^). The expressions for these variables are given as:

32 $$k=1.0635x{10}^{11}exp\left(\frac{-124850}{RT}\right)$$

33 $${K}_{{CO}_{2}}=9.099x{10}^{-7}exp\left(\frac{69691.8}{RT}\right)$$

34 $${K}_{{H}_{2}}=9.6104x{10}^{-4}exp\left(\frac{39942.0}{RT}\right)$$

35 $${K}_{eq}=exp\left[\left(\frac{28183}{{T}^{2}}+\frac{17430}{T}-8.2536log\left(T\right)+2.8032x{10}^{-3}T\right)+33.165\right]$$

## Results and discussions

### Materials characterization

#### Physicochemical characterization of the active phase

**Fig. 2:**
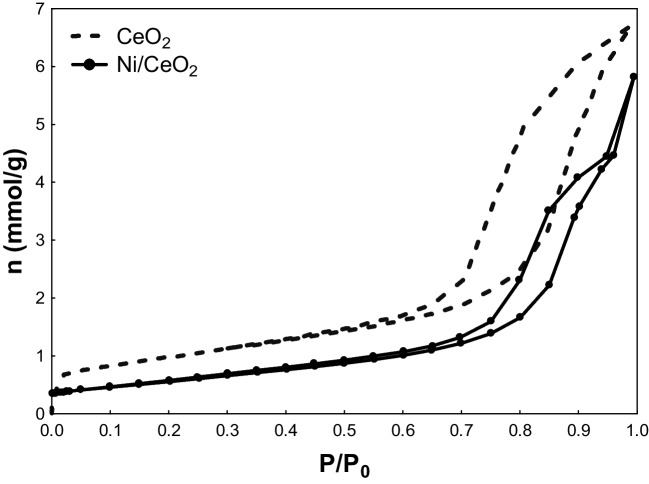
N_2_ adsorption − desorption isotherms at − 196 °C of CeO_2_ and Ni/CeO_2_-powdered catalyst

The specific surface area (*S*_BET_) and micropore volume (*V*_micro_) were determined by applying the Brunauer–Emmett–Teller and Dubinin-Radushkevich equations to the N_2_ isotherm, resulting in low surface areas. The volume of N_2_ adsorbed at *P*/*P*_0_ = 0.95 was considered as the total pore volume (*V*_total_), while the mesopore volume (*V*_meso_) was calculated by the difference between the *V*_total_ and *V*_micro_. As shown in Fig. 2, type IV isotherms were obtained for the CeO_2_ and Ni/CeO_2_ samples, which according to the IUPAC classification correspond to mesoporous materials. The isotherms present a low N_2_ uptake at low relative pressures, meaning they contain low micropore volume. As the relative pressures increased, the N_2_ uptake augmented, and a hysteresis loop is observed in both cases which is attributed to the presence of mesopores. It is seen that the hysteresis loop closes at high relative pressures due to the existence of wide mesopores, possibly produced during the calcination. Moreover, the addition of Ni on the CeO_2_ surface led to mesopores blockage, as shown with the diminution of *S*_BET_ and *V*_meso_ (see Table 1), suggesting that Ni particles are dispersed on the CeO_2_ surface blocking part of the porosity of the material.

**Table 1:** N_2_ sorption characterization of CeO_2_ and Ni/CeO_2_ powdered active phase

Sample	*S*_BET_ (m^2^ g^−1^)	*V*_total_ (cm^3^ g^−1^)	*V*_micro_ (cm^3^ g^−1^)	*V*_meso_ (cm^3^ g^−1^)
CeO_2_	78	0.21	0.04	0.17
Ni/CeO_2_	45	0.15	0.02	0.13

**Fig. 3:**
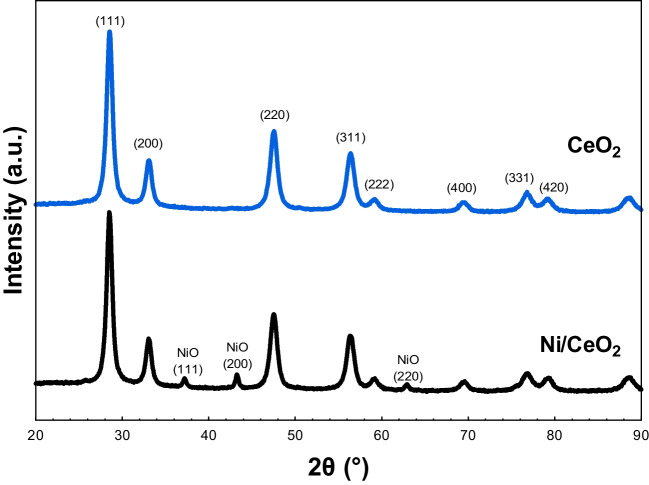
X-ray diffractograms of CeO_2_ support and Ni/CeO_2_ active phase

**Fig. 4:**
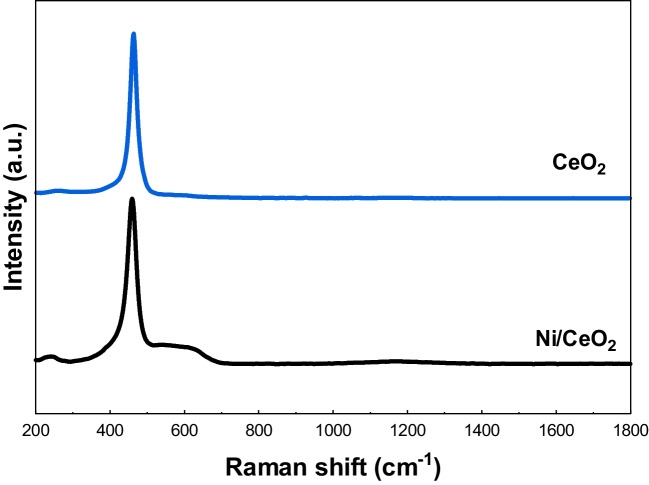
Raman spectra of CeO_2_ support and Ni/CeO_2_ active phase

The crystal structure of CeO_2_ and the catalytic compound was analyzed by X-ray diffraction and Raman spectroscopy, and the results are presented in Figs. 3 and 4, respectively. The X-ray diffractograms of both samples (Fig. 3) revealed the characteristic peaks of the fluorite face-centered cubic (FCC) crystalline structure of ceria (JCPDS 00–034-0394) at 28.5, 33.1, 47.6, and 56.5°, corresponding to the (111), (200), (220), and (311) planes (Tana et al. 2009). Besides, the Ni/CeO_2_ analysis showed additional little peaks at 37.2, 43.3, and 63° ascribed to (111), (200), and (220) crystallographic planes of the FCC of NiO (JCPDS 00–047-1049) (Nguyen et al. 2018). The lattice parameter of CeO_2_ calculated from XRD data was 0.5411 nm, which stays in accordance with the value reported in JCPDS 00–034-0394. The incorporation of nickel slightly displaced the CeO_2_ peaks in Ni/CeO_2_ active phase, which is also manifested in the increase of the lattice parameter of CeO_2_ to 0.5420, thus implying that some nickel cations are introduced into the crystal lattice of ceria. Instead, NiO particles are assumed to be mainly distributed on the CeO_2_ surface. The crystal sizes of CeO_2_ and Ni/CeO_2_ were obtained by the Scherrer equation (Scherrer 1912; Burton et al. 2009). The addition of nickel did not cause a significant change in the average size of the ceria crystals since the results were similar for both the pure CeO_2_ (8.77 nm) and the catalytic Ni/CeO_2_ (9.27 nm).

As plotted in Fig. 4, Raman spectra of pure ceria contains a single band at 463.9 cm^−1^, attributed to the F_2g_ mode of the fluorite-type cubic crystal structure of CeO_2_ (Rui et al. 2021; Kumaran et al. 2022; Lee). This peak corresponds to a symmetric Ce–O stretching vibration occurring around its equilibrium position in the octahedral sites of the cubic cell (Winter et al. 2019; Muduli and Ranjan Sahoo 2023; Lee 2017). Owing to the presence of nickel and its interaction with ceria, the F_2g_ peak shifted to 459.5 cm^−1^ in the spectra of Ni/CeO_2_ active phase (Muduli and Ranjan Sahoo 2023). Further, a small shoulder is present in the spectra of Ni/CeO_2_ catalyst between 500 and 660 cm^−1^, which is assigned to oxygen vacancies located at interfacial sites between Ni and CeO_2_ (Lin et al. 2023).

These results evidence insertion of Ni^2+^ cations into the CeO_2_ network replacing Ce^4+^ cations, and the oxygen vacancies are created to compensate the positive charge inbalance and relax crystal tensions due to the smaller size of the Ni^2+^ cations. The oxygen vacancies act as crucial cooperative sites in addition to metal active sites, since they bind adsorbates more strongly than normal oxide sites, promoting the dissociation (Xu et al. 2021; Li et al. 2022). In conclusion, Ni/CeO_2_ characterization by N_2_ adsorption, XRD, and Raman spectroscopy evidence the formation of NiO particles on the CeO_2_ surface and inserption of Ni^2+^ cations into the CeO_2_ framework. High catalytic activity of this active phase for CO_2_ methanation has been related with the presence of two type of active sites: oxygen vacant sites associated to the Ni^2+^-CeO_2_ entities that are efficient for CO_2_ chemisorption and dissociation, and metal Ni, obtained upon surface NiO nanoparticles reduction, which is expected to dissociate H_2_.

**Fig. 5:**
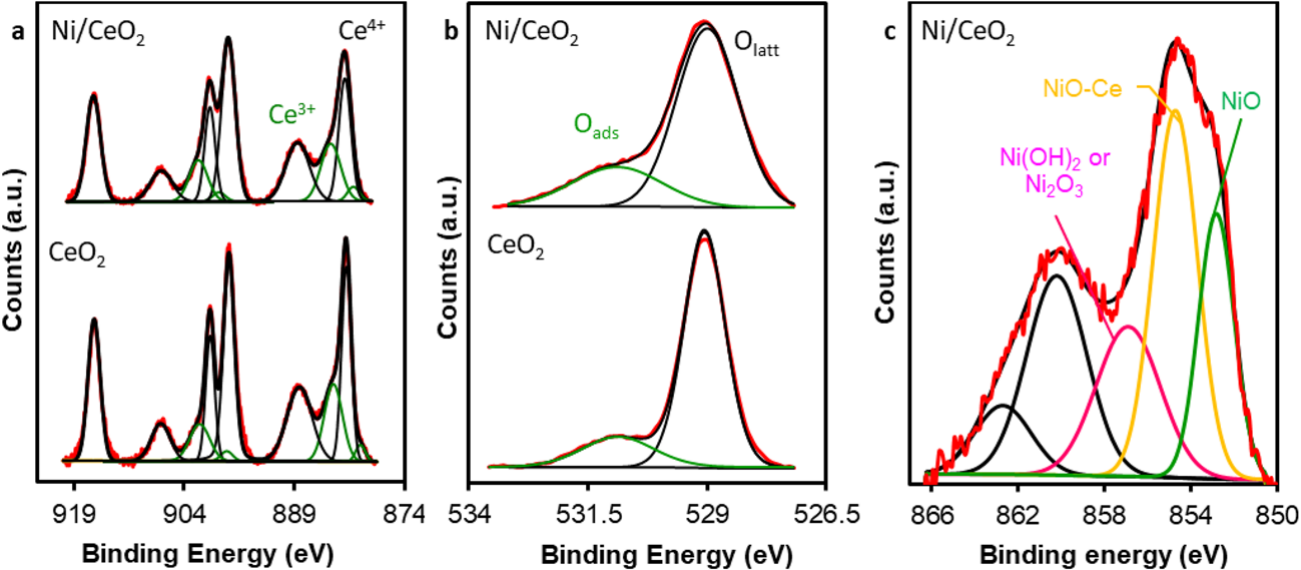
XPS results: **a** Ce_3d_, **b** O_1s_, and **c** Ni_2p3/2_ regions

The surface chemistry of the Ni/CeO_2_ active phase and CeO_2_ support was analyzed by XPS. Ce_3d_, O_1s_, and Ni_2p_ XPS spectral regions are shown in Fig. 5. Five doublets are required to deconvolve the Ce_3d_ region (Fig. 5a). The peaks pairs at 882.0–900.3 eV, 888.2–907.0 eV, and 898.0–916.3 eV are attributed to Ce^4+^ whereas the peaks at 880.3–898.6 eV and 884.0–902.3 eV are assigned to Ce^3+^. The proportion of Ce^3+^ detected by XPS must be related with the reducibility of the surface. Sellers-Antón et al. (2020) show that the reducibility of the surface is improved by the addition of Ni, mainly if this Ni is incorporated during the synthesis due to the higher dispersion and Ce-Ni contact in this case. Consequently, it is expected that the amount of Ce^3+^ detected by XPS increases in Ni/CeO_2_ active phase regarding CeO_2_ support. The surface Ce^3+^ content obtained by XPS was 18.9 and 22.5 for CeO_2_ and Ni/CeO_2_, respectively, due to the improved surface reducibility which confirm the successful synthesis of Ni/CeO_2_. This effect must be also observed in the O_1s_ region. The O_1s_ region requires two peaks for deconvolution (see Fig. 5b): one peak centered at 529.0 eV, associated with surface lattice oxygen (*O*_latt_), and another peak centered at 531.0 eV, attributed to adsorbed oxygen species (*O*_ads_). Typically, *O*_ads_ species are present at oxygen vacancies, so a higher concentration of *O*_ads_ species suggests a greater population of oxygen vacancies. Therefore, the ratio of surface *O*_ads_ to *O*_latt_ can serve as an indicator of surface reducibility. The *O*_ads_/*O*_latt_ ratio was 0.22 and 0.32 for CeO_2_ and Ni/CeO_2_, respectively indicating that Ni improves the surface ceria reducibility. Finally, the Ni_2p3/2_ region of active phase is depicted in Fig. 5c. In this spectral region, a primary band is observed within the 851–859 eV range, accompanied by a satellite structure at higher binding energies (859–866 eV). To deconvolute the Ni_2p3/2_ region, three peaks are necessary, occurring around 853.2, 855.0, and 857.2 eV. These peaks correspond to surface Ni^2+^ species within the NiO structure (surface NiO species), Ni^2+^ species closely associated with the ceria surface (NiO-Ce species), and Ni^3+^ or Ni(OH)_2_ species, respectively (Sellers-Antón et al. 2020; Cárdenas-Arenas et al. 2020).

#### Characterization of ceramic catalysts

**Fig. 6:**
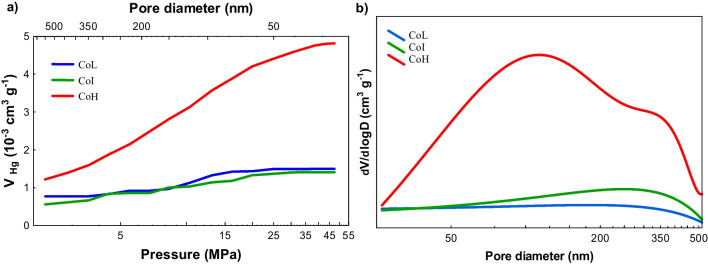
Results of mercury porosimetry of the three different cell densities cordierite monoliths: **a** cumulative intrusion curves and **b** pore size distribution

To determine the meso and macro porosity of the bare monolithic materials, mercury porosimetry characterization was performed. The pore volume and pore size distribution are presented in Fig. 6a and b, respectively. Results corroborated the low porosity of cordierite monoliths; however, a significant difference is observed in the CoH material regarding the CoL and CoI. The porosity profile of CoH monolith (Fig. 6a) shows a larger increment of the intrusion volume in the macropores range up to mesopores of about 26 nm. Besides, a wider distribution of pore diameter was found in this material (Fig. 6b).

**Fig. 7:**
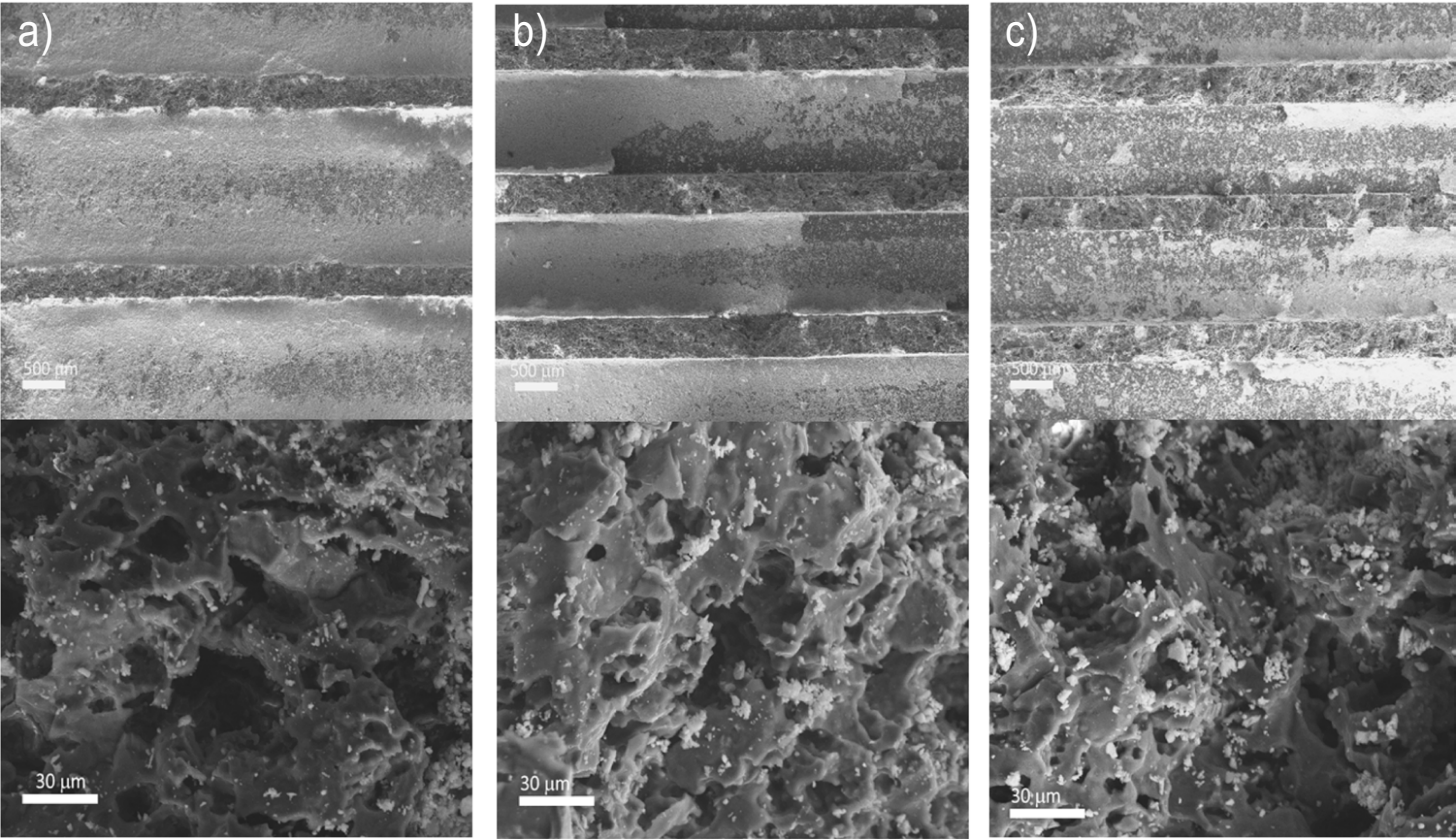
SEM micrographs of cordierite monoliths loaded with Ni/CeO_2_: **a** CoL, **b** CoI, and **c** CoH. Top images, monolith channels; down images, channel walls

SEM analysis was performed to observe the morphology and active phase distribution on the cordierite monoliths (Fig. 7a–c). It is observed that the surfaces of cordierite monolith walls are rough, exhibiting some irregular pores with sizes on the order of macropores, which is consistent with the Hg-porosimetry results and has also been observed in this type of material by other authors (Brussino et al. 2016; Tang et al. 2021). Their low porosity leads to a limited diffusion of the active phase during the impregnation; therefore, most of the load is concentrated on the surface of the channels, especially for monoliths CoL and CoI (Fig. 7a and b).

**Fig. 8:**
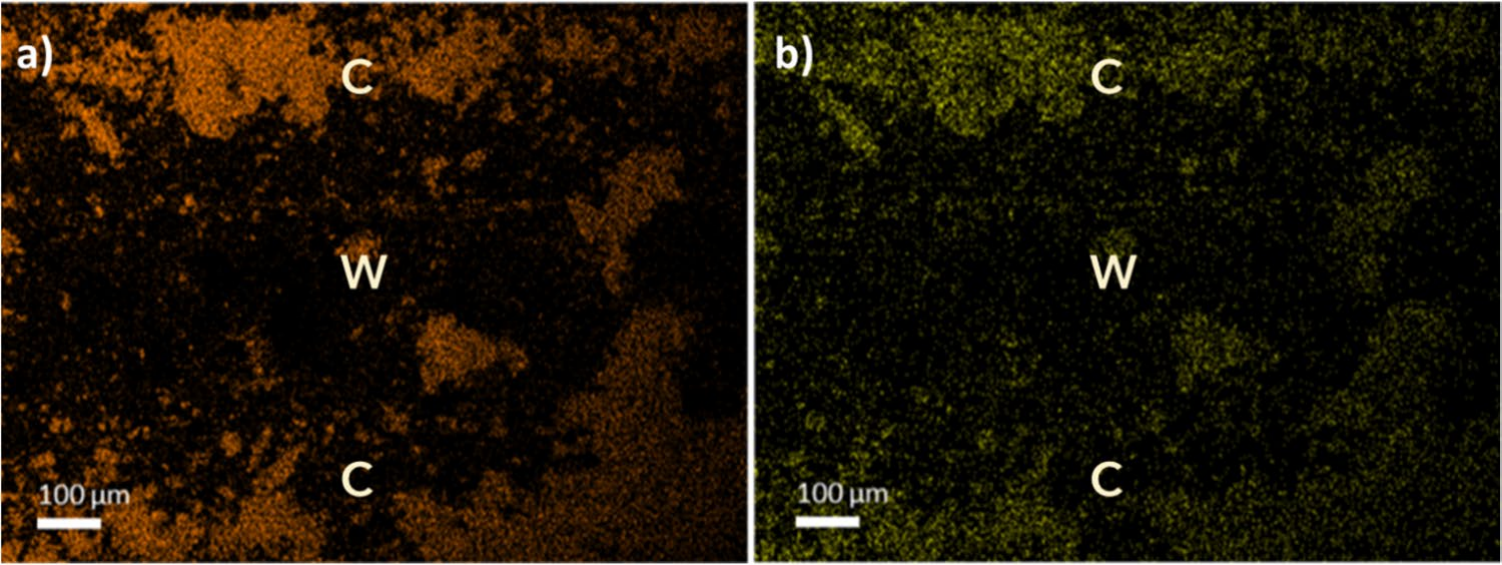
Chemical mapping of the active phase Ni/CeO_2_ deposited in the CoH ceramic monolith within the channels (C) and walls (W): **a** Ce and **b** Ni

It is also evident that, in the case of the low cell density, the active phase layer results thicker than in the other two monoliths due to the smaller exposed surface available for deposition. Notwithstanding, the bigger pore volume of CoH monolith resulted in a higher diffusion of the Ni/CeO_2_ ethanolic solution during impregnation, thus, in a higher amount of the active phase introduced into the channels walls (as depicted in Fig. 7c). This was also corroborated by chemical mapping (see Fig. 8), where it can be observed that the active phase is not only uniformly distributed along the channels but also within their walls. Cerium (Fig. 8a) and nickel (Fig. 8b) were detected inside the walls in the case of the CoH monolith. Furthermore, it is essential to note that during the preparation of samples for SEM analysis, part of the active phase coating, especially in low and intermediate cell densities, detached when cutting them. This separation can be attributed to its concentration in the walls and the poor attachment resulting from the low porosity.

**Fig. 9:**
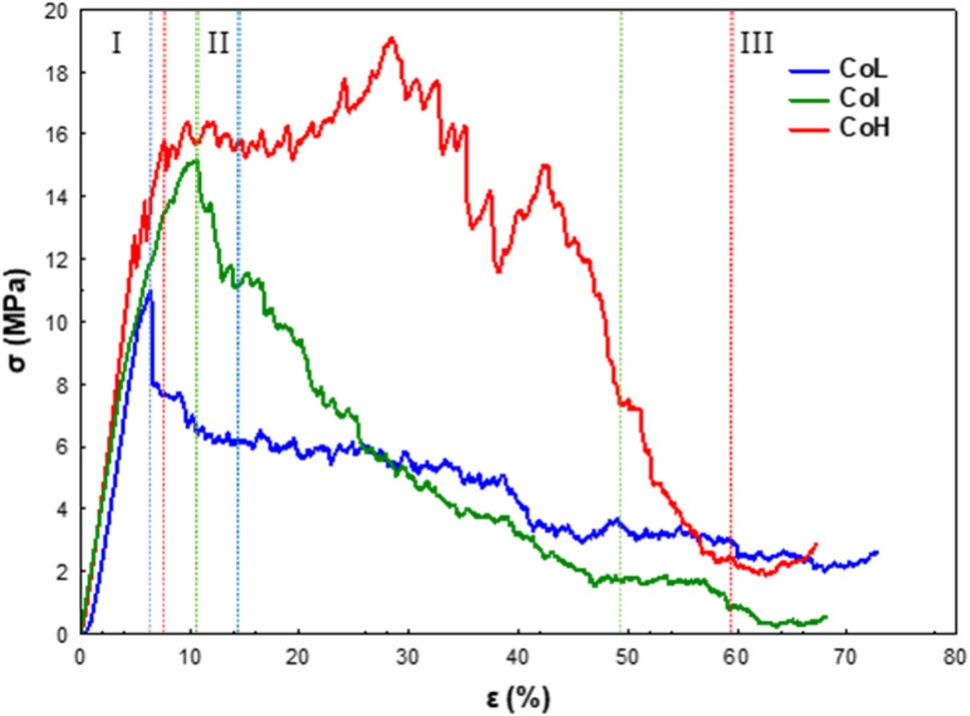
Stress–strain curves of cordierite monoliths of different cell densities

The mechanical properties of the monoliths with different cell densities were evaluated through stress–strain curves (Fig. 9) obtained by compression along the channels. The maximum compressive stress values achieved were 10.96, 15.15, and 19.10 MPa for the low, intermediate, and high cell densities, respectively (Table 2). These results are in accordance with the amount and thickness of the channels, which contribute to greater mechanical strength.

**Table 2:** Maximum compressive stress (*σ*_max_) and Young’s modulus (*E*) of cordierite monoliths obtained from stress–strain curves

Muestra	*σ*_máx_	*E*
	MPa	MPa
CoL	10.96	135
CoI	15.15	158
CoH	19.1	210

The deformation process can generally be classified into three regions (I, II, and III) based on the compressive stress–strain behavior. The first region (I) comprises the almost linear increase in compressive stress as the strain increment occurs. This behavior is attributed to the elastic buckling of cell walls. Afterwards, the stress–strain curve reaches maximum stress, and a rapid drop in the compressive stress appears in region II. This rapid drop results from the fast transition of elastic buckling to plastic buckling of cell walls, followed by debonding fractures at cell/cell interfaces (Lee et al. 2002). For the case of high cell density, a slight plateau region is appreciated, which may indicate a viscoelastic behavior (Dorado et al. 2023). The stress maintains a roughly constant value lower than the maximum compression stress in region III, where a continuous fracture of the cell walls was observed.

### Catalytic results

**Fig. 10:**
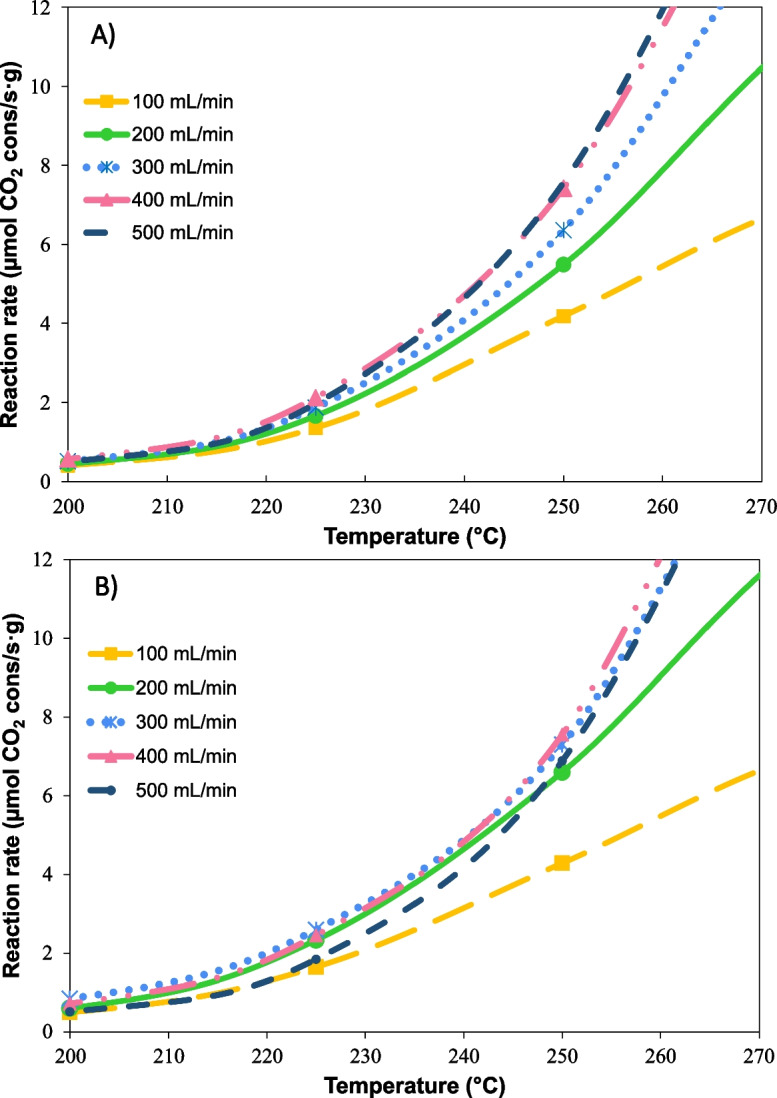

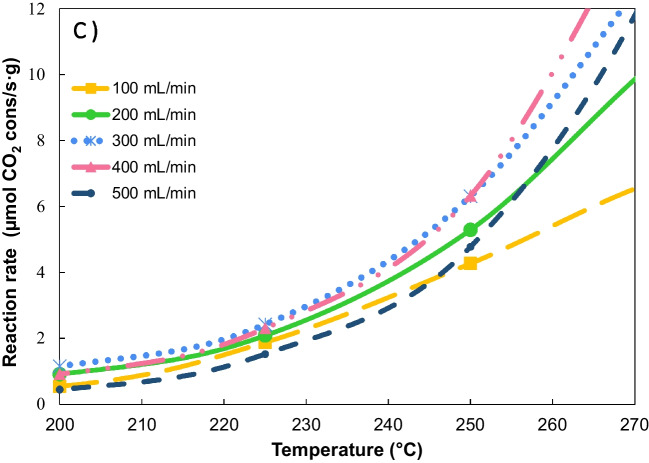
Reaction rate normalized by weight of Ni/CeO_2_ supported on commercial cordierite monoliths of different cell densities: **a** ~ 200 cpsi, **b** ~ 400 cpsi, and **c** ~ 500 cpsi

The catalytic performance of the Ni/CeO_2_-loaded cordierite monoliths was analyzed in the CO_2_ methanation under 10% CO_2_ and 40% H_2_ in Ar balance, at total flows varying from 100 to 500 mL min^−1^. The reaction rates normalized by weight of active phase are depicted in Fig. 10. As exhibited in Fig. 10a, the low cell density monolith works under diffusional control at flows below 400 mL min^−1^. In contrast, attributed to higher active phase dispersion, this behavior is observed up to 300 mL min^−1^ for intermediate and high cell density monoliths (Fig. 10b and c), where chemical control is achieved. However, flow rates higher than 400 mL min^−1^ cause a decrease in the reaction rate. Under diffusional control, an increment in volumetric flow enhances the reactants supply to the catalyst and facilitates the withdrawal of the products. Conversely, excessively high injection rates force the gas to flow inside the straight channels, leading to a laminar flow regime. In this regime, the flow is non-uniform because of the friction with the walls, resulting in significantly higher velocities in the center of the channel compared to the proximity of the walls (Kundu et al. 2016; Caenn et al. 2017). Consequently, the interaction with the catalyst walls is diminished and, thus, the effectiveness is also reduced.

The effect of the flow rate was more significant in monoliths with higher cell densities, which may be promoted by their lower open frontal area speeding the reagents flow. The open frontal area refers to the part of the cross-section of the monolith that is free for the flow of gas and depends on the cell density, wall thickness, and washcoat layer (Cornejo et al. 2021). Owing to the characteristics of the cell density (surface to volume ratio ~ 45 regarding ~ 20), the CoI and CoH monoliths achieve better distribution of Ni/CeO_2_ than CoL. However, when the reactive mixture is supplied to these materials, it is further induced to flow inside the channels, interacting less with the active phase.

The effect of the flow rate was observed in the CO_2_ conversion obtained with different cell densities (Figure S1). For the CoL monolith (Figure S1a), increasing the flow rate from 100 to 200 mL min^−1^ results in a higher minimum temperature required for the reaction to take place; though, rising from 300 to 500 mL min^−1^ does not represent a significant change in the conversion of CO_2_. In contrast, Figure S1b shows that CoH monolith is more affected by gas flow rate changes. For the CoH monolith, larger flow rates clearly affect the performance of the catalyst, requiring up to 50 °C more to treat 500 mL min^−1^ compared to 100 mL min^−1^. The increase in the minimum reaction temperature may be attributed to both the introduction of the active phase in the channel’s walls (as observed in SEM) and the reduction of diffusion caused by the straight design. These factors together result in poor contact between the reactive gas mixture and the Ni/CeO_2_, which makes part of the active phase inaccessible and unusable, principally that inside the walls.

**Fig. 11:**
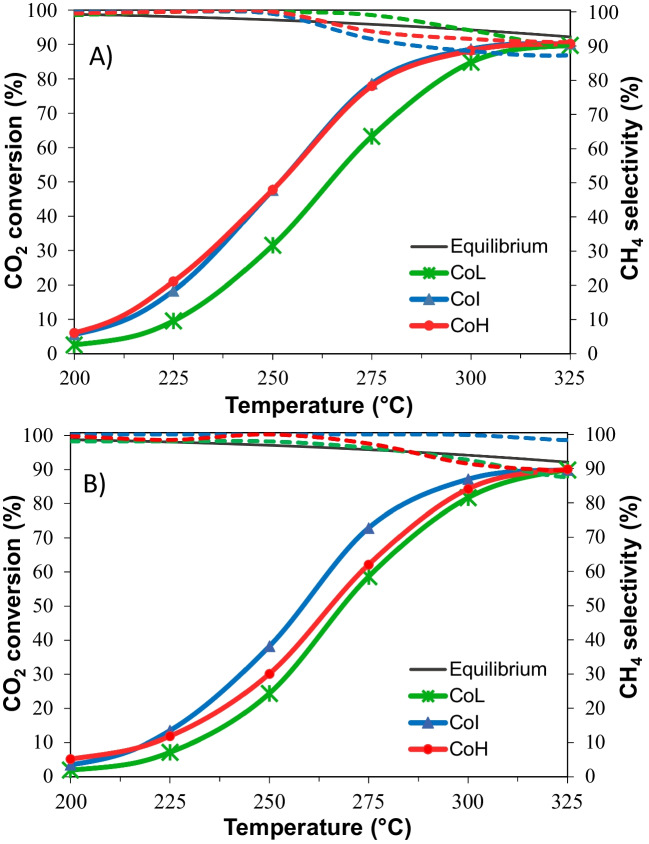
CO_2_ conversion (continuous line) and selectivity to CH_4_ (dashed line) obtained for CO_2_ methanation with cordierite monoliths of different cell densities considering the same gas hourly space velocity: **A** GHSV ≈ 800 h^−1^ and **B** GHSV ≈ 1500 h^−^.^1^

Further, the catalytic activities of the three cordierite monoliths using similar gas hourly space velocities were assessed (Fig. 11). It is evident that higher cell densities exhibit improved catalytic activity, this is associated with their greater exposed surface area in which the Ni/CeO_2_ is distributed, and the gases can react. In the case of lower GHSV (Fig. 11a), both CoI and CoH monoliths displayed similar performances, achieving superior CO_2_ conversion (about 15%) and reaching total conversion at a lower temperature (around 16 °C less) compared to CoL. Although CoH has a slightly bigger surface to volume ratio in relation to CoI (44.9 and 42.3, correspondingly), and thus better activity could be expected, the Ni/CeO_2_ introduced inside its channels walls resulted useless and reduced the benefit of CoH configuration. This effect is more prominent when employing higher GHSV (Fig. 11b). As the reagents flow increased, diffusion through the walls diminished to a greater extent, leading to a notable reduction in the catalytic activity in the CoH material.

On the other hand, a slight decrease in the selectivity to CH_4_ is observed, which may be related to the temperature reached over the catalyst. The high catalyst surface-to-bulk gas volume ratio results in a great rate of heat produced over the catalyst surface and heat transferred to the gas phase (Barbato et al. 2015). Therefore, the surface may get hotter with the potential formation of hot spots, which in turn, can activate the reverse water–gas shift reaction (RWGSR) (De Piano et al. 2022). This explains the increase of conversion, while obtaining slightly lower selectivity.

### Fluid dynamic study

**Fig. 12:**
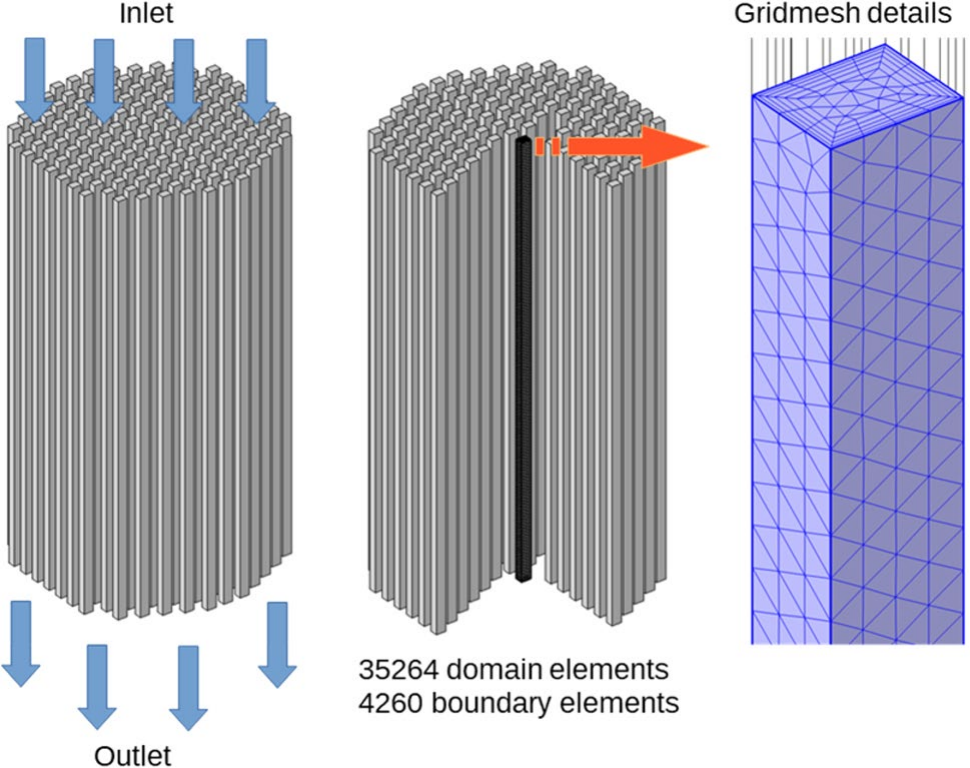
Computational geometry of the monolithic catalyst and the triangular gridmesh used for one channel

The mathematical model formulated in Eqs. (5) to (28) was numerically solved using the software COMSOL Multiphysics. This software utilizes advanced numerical routines for solving coupled partial differential equations through the finite element method. For this method, the geometrical domain is divided into multiple subdomains, where the unknown variables are calculated at each time step. Figure 12 shows the geometric representation of the monolithic catalyst and the gridmesh used in one of the channels.

**Fig. 13:**
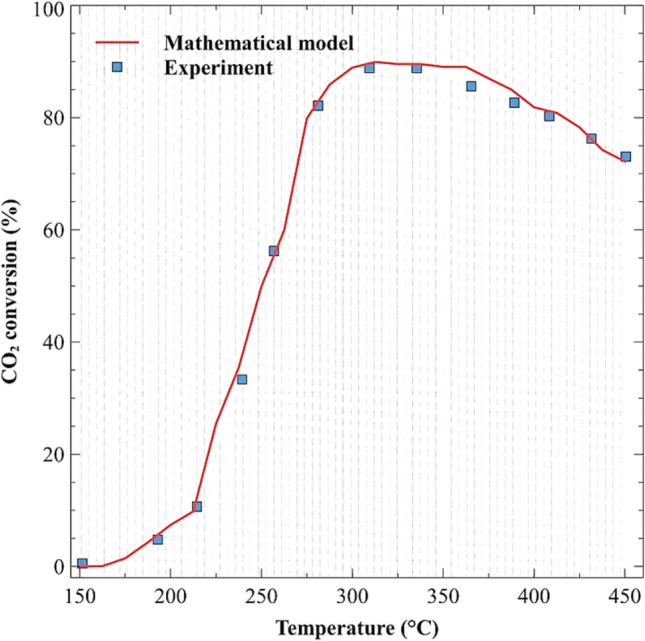
Comparison of CO_2_ conversion calculated via the numerical solution of the mathematical model with the experimental data obtained in the laboratory for the CoL monolith (200 cpsi) treating a volumetric flow of 100 mL min^−^.^1^

Due to the simple geometry of the straight channels, the numerical solution of the mathematical model can be performed in just one of the channels, under the assumption that mass and momentum transport, as well as the chemical reaction rates, are equal in all the channels. We verified that the gridmesh containing 35,264 triangular elements is enough to yield accurate and consistent solutions of the mathematical model. Once the mathematical model is programmed and solved in COMSOL Multiphysics, its predictability is assessed by comparing the CO_2_ conversion (see Figs. 13 and S2). According to the curves plotted in Fig. 13, the mathematical model solution shows good agreement with the experimental results considering the monolith of 200 cpsi and a gas injection rate of 100 mL min^−1^. The error, quantified as the area between the curves plotted in Fig. 13, is approximately 313.4%/°C.

**Fig. 14:**
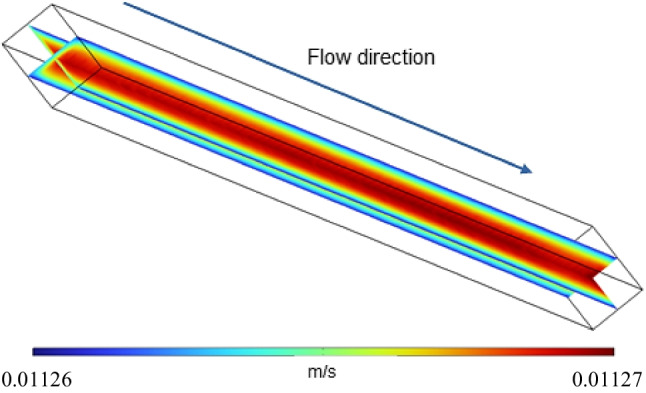
3D velocity profile of the reactive gas mixture inside one of the channels of the CoL monolithic catalyst at a flow of 200 mL min^−^.^1^

The velocity profile of the gas mixture inside the monolith channels is of great importance to understand the efficiency of supplying reactants to the reactive sites and removing the products through the gas stream. For this purpose, the fluid velocity achieved in the CoL monolith for a 200 mL min^−1^ gas injection is depicted in Fig. 14. The velocity profile rapidly acquires a stationary state, and therefore, it is not necessary to analyze the velocity at each time step. In general, a radial profile is developed on the transverse section of the channel, and such profile is kept from the entrance to the exit. As expected, the maximum velocity is found at the center of the channel. The absence of vortices is observed, which could eventually hinder the diffusion of the gaseous reagents into the monolithic structure. Nevertheless, this diffusion can be enhanced by designing monoliths with tortuous channels that promote turbulence within the channels (Aguilar-Madera et al. 2021). The maximum velocity achieved when injecting 200 mL min^−1^ is about 0.01127 m s^−1^.

This behavior was also corroborated in the CoH monolith at different inlet flows (see Figure S3). In Figure S3, it is also noticeable that for the smaller channel (CoH), the same increase in reagent flow (100 mL min^−1^) produces a higher rise in fluid velocity (around 2 times) compared to the larger channel of CoL monolith (approximately 1.5 times). This augment in velocity leads to lower diffusion through the CoH channel walls. Consequently, the interaction and use of the deposited active phase mainly in the walls are reduced, supporting the previously mentioned catalytic results.

**Fig. 15:**
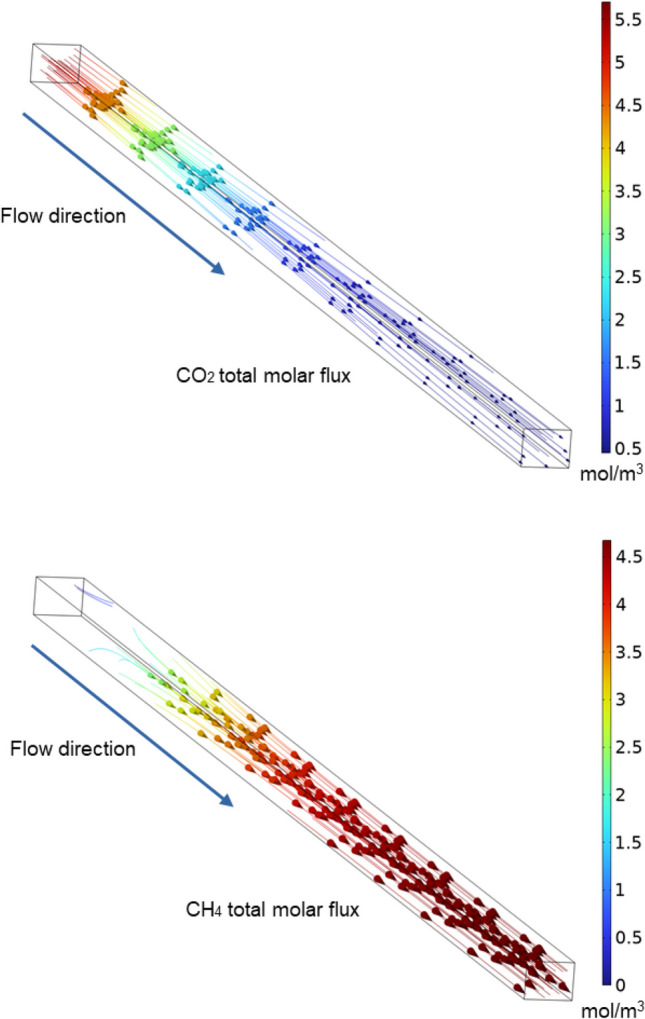
Molar concentration and main trajectories of the CO_2_ and CH_4_ in one channel of the CoL monolithic catalyst. Flow rate of 100 mL min^−1^ at 350 °C

The local concentrations of CO_2_ and CH_4_ inside the channel of the CoL monolith with a flow rate of 100 mL min^−1^ were estimated and graphed in Fig. 15. In this figure, we have selected the time when temperature reaches 350 °C, which are the conditions for maximum CO_2_ conversion. The maximum methane concentration reaches 4.5 mol m^−3^, while the maximum concentration of CO_2_ is that of feed conditions near the entrance, and eventually, it drastically diminishes through the monolith channel. In the analyzed case, most of the methane is produced from the middle and towards the exit of the monolith, while CO_2_ is simultaneously consumed. In general, both chemical species develop linear streamlines along the monolith channel.

**Fig. 16:**
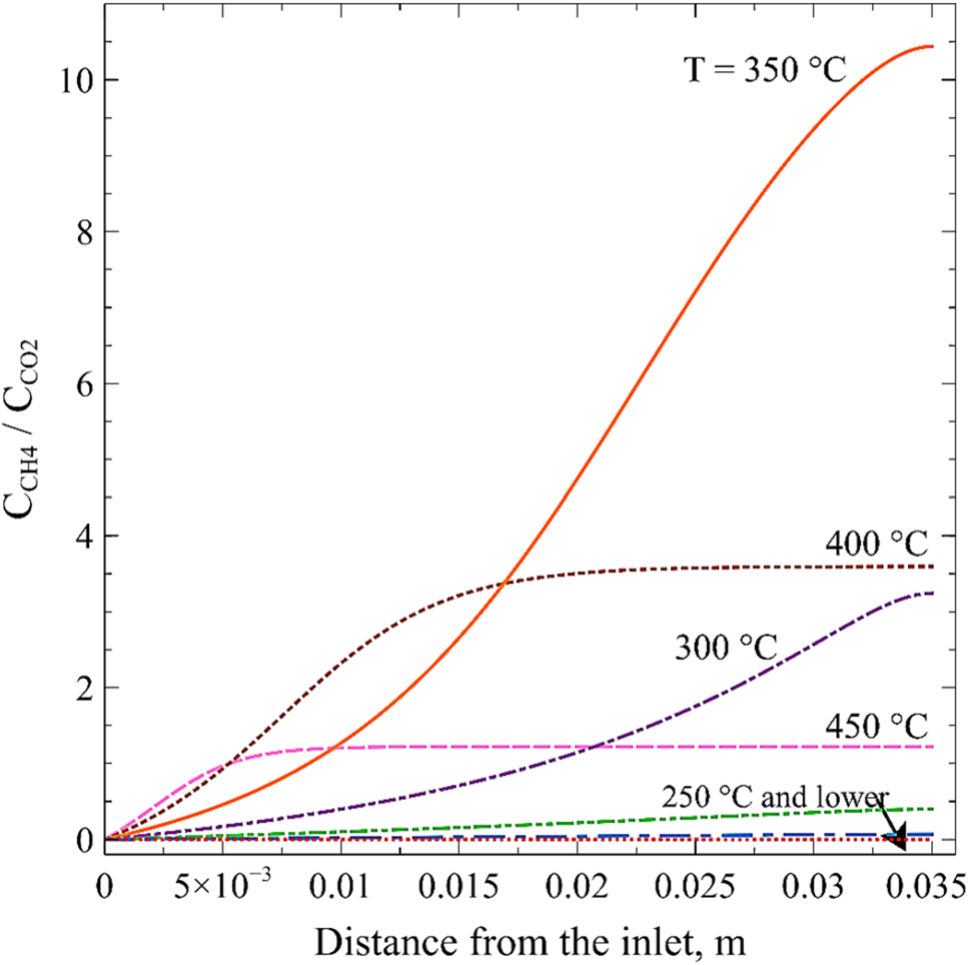
Ratio of CH_4_ to CO_2_ concentrations assessed at the center of the channel of the 200 cpsi monolith with a volumetric flow rate of 100 mL min^−^.^1^

Finally, Fig. 16 shows the variation of the ratio of CH_4_ to CO_2_ concentrations along the channel, evaluated at the center of the channel, for various temperatures. As observed, there is one entrance length of about 5 mm where the methane commences to be produced significantly. As temperature rises to 350 °C, methane is massively produced throughout most of the monolith, mainly near the exit. The maximum concentration ratio reached, approximately 10, is achieved at 350 °C. It is also evident that at higher temperatures, the higher CH_4_/CO_2_ ratio (lower than 10) is reached closer to the entrance (450 °C) and the middle (400 °C), after which concentrations remain constant. Therefore, we can affirm that the 3.5-cm-long monolith is proper for working at the lowest temperature that allows achieving higher conversion without misusing the catalyst.

## Conclusions

The influence of the cell density configuration of monolithic catalysts was investigated in the Sabatier reaction for CO_2_ conversion using cordierite monoliths. Ni/CeO_2_ was selected as the active phase, which was loaded on commercial cordierite monoliths of three different cell densities (200, 400, and 500 cpsi) by means of immersion into an ethanolic suspension. The low porosity of the CoL and CoI materials led to concentration of the active phase on the channels’ surface due to a limited diffusion of the ethanolic suspension through the structure. However, in the case of the CoH monolith, owing to a larger pore volume, some Ni/CeO_2_ was deposited into the channel’s walls, as observed in SEM and EDS micrographs.

The catalytic evaluation indicated diffusional control at flows below 500 mL min^−1^ for the CoL monolith, whereas CoI and CoH materials achieved chemical control at flows above 300 mL min^−1^. Though, larger increase in volumetric flow in CoI and CoH materials led to a decrease in reaction rate since their lower open frontal area speeded the reagents flow through the channels, reducing the interaction with the catalysts. Regarding the CO_2_ conversion, the three materials were analyzed at the same GHSV. Results confirmed the benefit of attaining a better distribution and availability of Ni/CeO_2_ by means of the bigger surface to volume ratio in high cell densities. Notwithstanding, despite the greater exposed surface area of CoH monolith, the introduction of active phase into the walls repercussed on its accessibility. Therefore, CO_2_ conversion was reduced at high GHSV, in which the reagents’ diffusion through the ceramic material was further diminished; thus, the active phase introduced into the walls was misused.

Furthermore, in the present work, a comprehensive mathematical model was formulated and successfully solved through COMSOL Multiphysics software to simulate the reaction of methanation of CO_2_. The model exhibited acceptable convergency with the experimental data and helped to study the fluid dynamics of the monolithic support as well as the velocity profile within the channels. In this sense, a radial profile, with the maximum velocity of the fluid at the center of the channel, is formed close to the inlet and kept to the outlet. Additionally, an entry length of about 0.5 cm was observed when analyzing the concentration of CO_2_ and CH_4_. Moreover, it was observed a maximum production of 4.5 mol m^−3^ of CH_4_ at 350 °C utilizing the whole monolith length. Lastly, the streamlines of chemical species were linearly established along the channel.

Overall, the cell density is an important factor controlling the catalytic activity, but the control of the Ni/CeO_2_ distribution on the porosity of the monolith is also mandatory to optimize the active phase catalytic performance since repercussed on its accessibility. Hence, the insights gained from this study hold great promise for advancing and refining monolithic catalysts used in CO_2_ methanation, contributing to the pursuit of sustainability and environmental conservation objectives.

## Supplementary Information

Below is the link to the electronic supplementary material.

Supplementary file1 (DOCX 374 KB)

## Data Availability

Data and materials are available from the corresponding author on reasonable demand.
