# Supplementary material for: Modeling and experimental analysis of CO2 methanation reaction using Ni/CeO2 monolithic catalyst
Source: Environ Sci Pollut Res Int. 2024 Apr 25;31(22):32766–83. doi: 10.1007/s11356-024-33327-5 (PMC11512854; doi:10.1007/s11356-024-33327-5)
Supplement: Supplementary file 1 — Supplementary file1 (DOCX 374 KB) [file 11356_2024_33327_MOESM1_ESM.docx]

**MODELING AND EXPERIMENTAL ANALYSIS OF CO2 METHANATION REACTION USING Ni/CeO2 MONOLITHIC CATALYST**

**A. Parra-Marfil ^1,2^, R. Ocampo-Pérez^2*^, C.G. Aguilar-Madera^3^, F. Carrasco-Marín^1^, A.F. Pérez-Cadenas^1^, A. Bueno-López^4^, E. Bailón-García^1*^**

*^1^* *Materiales Polifuncionales Basados en Carbono (UGR-Carbon), Dpto. Química Inorgánica - Unidad de Excelencia Química Aplicada a Biomedicina y Medioambiente - Universidad de Granada (UEQ-UGR), ES 18071-Granada, España.*

*^2^* *Centro de Investigación y Estudios de Posgrado (CIEP), Facultad de Ciencias Químicas, Universidad Autónoma de San Luis Potosí (FCQ-UASLP), MX 78260-San Luis Potosí, México.*

*^3^**Facultad de Ciencias de la Tierra, Universidad Autónoma de Nuevo León (UANL), Carretera a Cerro Prieto Km. 8 Ex Hacienda de Guadalupe, MX 67700-Linares, México.*

*^4^Dpto. de Química Inorgánica, Universidad de Alicante (UA), ES 03080-Alicante, España.*

**Figure S1.** CO_2_ conversion obtained with Ni/CeO_2_-loaded commercial cordierite monoliths of: a) ~200 cpsi; and b) ~500 cpsi.

**Figure S2.** CO2 conversion obtained with Ni/CeO2-loaded commercial cordierite monoliths with 200 cpsi and 500 cpsi for different gas flows: a) calculated via the numerical solution of the mathematical model; and b) experimental data.


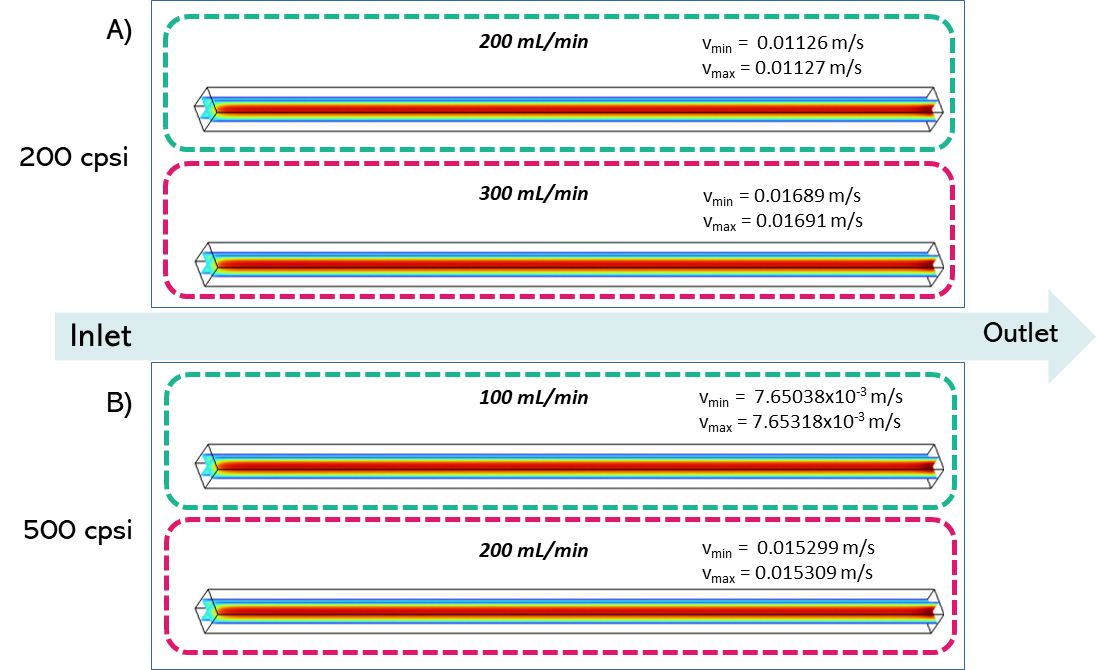


**Figure S3.** Comparison of the 3D velocity profiles of the reactive gas mixture at different flows to achieve similar gas hourly space velocities (green box GHSV ≈ 800 h^-1^, and pink box GHSV ≈ 1500 h^-1^) inside one of the channels of: a) CoL monolith; and b) CoH monolith.
